# Flow-Based Cytometric Analysis of Cell Cycle via Simulated Cell Populations

**DOI:** 10.1371/journal.pcbi.1000741

**Published:** 2010-04-15

**Authors:** M. Rowan Brown, Huw D. Summers, Paul Rees, Paul J. Smith, Sally C. Chappell, Rachel J. Errington

**Affiliations:** 1 School of Medicine, Cardiff University, Cardiff, United Kingdom; 2 Multidisciplinary Nanotechnology Centre, School of Engineering, Swansea University, Swansea, United Kingdom; University of Washington, United States of America

## Abstract

We present a new approach to the handling and interrogating of large flow cytometry data where cell status and function can be described, at the population level, by global descriptors such as distribution mean or co-efficient of variation experimental data. Here we link the “real” data to initialise a computer simulation of the cell cycle that mimics the evolution of individual cells within a larger population and simulates the associated changes in fluorescence intensity of functional reporters. The model is based on stochastic formulations of cell cycle progression and cell division and uses evolutionary algorithms, allied to further experimental data sets, to optimise the system variables. At the population level, the *in-silico* cells provide the same statistical distributions of fluorescence as their real counterparts; in addition the model maintains information at the single cell level. The cell model is demonstrated in the analysis of cell cycle perturbation in human osteosarcoma tumour cells, using the topoisomerase II inhibitor, ICRF-193. The simulation gives a continuous temporal description of the pharmacodynamics between discrete experimental analysis points with a 24 hour interval; providing quantitative assessment of inter-mitotic time variation, drug interaction time constants and sub-population fractions within normal and polyploid cell cycles. Repeated simulations indicate a model accuracy of ±5%. The development of a simulated cell model, initialized and calibrated by reference to experimental data, provides an analysis tool in which biological knowledge can be obtained directly via interrogation of the *in-silico* cell population. It is envisaged that this approach to the study of cell biology by simulating a virtual cell population pertinent to the data available can be applied to “generic” cell-based outputs including experimental data from imaging platforms.

## Author Summary

One of the key challenges facing cell biologists today is understanding the influence of molecular controls in shaping and controlling cell growth and proliferation. There is growing recognition that abnormal progression through the cell cycle and the associated effects on the growth of cell populations has a major impact on a wide range of biological and clinical problems, including: tumour growth, developmental control, origins of chromosomal instability and drug resistance. Multiparameter flow cytometry is frequently used to assess proliferation dynamics of cellular populations using fluorescent reporters generating large data sets that can inform simulation models. We have developed stochastic computing approaches allied to evolutionary algorithms to produce simulated cell populations—providing a new approach to the analysis of real multi-variate data sets obtained by flow cytometry. The methodology delivers new insight on biological processes in delivering a continuous simulation of the dynamic evolution of a cellular system between fixed sampling points, hence, converting static data into dynamic data revealing the effective traverse of the cell cycle, restriction points and commitment gateways. The approach also permits the visualisation of the variation between individual cells reflecting biological heterogeneity and potentially Darwinian fitness, given that the simulation delivers a report on population dynamics in which each and every cell can be tracked.

## Introduction

Multiparameter flow cytometry is widely used to study the cell cycle and its perturbation in the context of both basic research and in routine clinical analysis [1]–[6]. Such analyses may use a wide range of fluorescent reporters that correlate to the expression of key molecular components of the cell cycle, such as cyclins and cyclin dependent kinases (CDK), [1] or quantify DNA content [5]. Regardless of the particular fluorophores used the quantitative methodology and the ensuing synthesis of biological knowledge is based on statistical analyses of the experimental data sets. For single variable distributions these may include calculations of moments of increasing orders to provide the mean, variance, skewness etc. or cumulative indices such as the Kolmogorov-Smirnov (K-S) test [7]–[9]. More complex, multi-variate approaches may involve discriminant function, cluster or principal component analysis in an *n*-dimensional space [10]–[12]. In all of these approaches there is a common procedural thread: acquisition of data is followed by a statistical parameterisation of the measurement set to which biological form or function can be correlated. In this work, we present an alternative, based on computational simulation of the experiment. A stochastic simulation of the cell cycle dynamics within a large population is initialised with reference to a flow cytometry data set and then evolved, using evolutionary computer algorithms, with assessment of fitness measures derived from comparisons to subsequent data sets. The cell-cycle information is then read directly from the *in-silico* populations.

The development of a simulated cell population approach has been driven by a requirement to track the evolution of large numbers of cells over multiple generations through the cell cycle and provide a means to track progression of both the whole cell population and distinct sub-groups [13],[14]. This is in the context of mapping the heterogeneity of cell cycle response to perturbation events e.g. effects on cell proliferation of anticancer therapeutics designed to block cell division. In this report we present the conceptual basis of this simulated cell cytometry and detail of the methodology adopted. To demonstrate the application of the technique and validate its potential we use it to quantify cell cycle perturbation in a tumour cell line by a topoismerase II inhibitor which causes endocycle routing in the late cell cycle.

**Figure 1:**
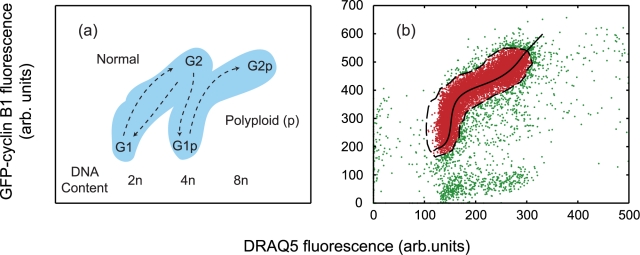
Plots indicating cell pathways through the cell cycle. (a) A schematic indicating cell cycle routing of cells treated with ICRF-193 in fluorescence space (GFP-cyclin B1 signal as a function of DRAQ5 signal). In control conditions U-2 OS cells divide and remain in normal cycle (representing the proliferative fraction), with ICRF-193 treatment the U-2 OS cells continue to cycle however they bypass mitosis to enter a polyploid cycle (p) (representing the non-proliferative fraction). (b) Segmentation of experimental flow cytometry data representing molecular expression and DNA content used to initialise the CPM. Experimental data at t_expt_ = 0: experiment non-gated data (green and red markers), experimental gated data/initial virtual population (red markers) and the dashed black line refer to the contour defining the gated data. The solid black line indicates the best representation of the fitted population (see text).

The aim of the simulation is to predict the dynamic evolution of a large population of *virtual* cells (*v*cells) through a life cycle corresponding to that prescribed by their real-life counterparts (as reported via flow cytometry experiments). Furthermore, the model seeks to account for perturbations in the cell cycle progression of the *virtual* population (*v*population). The spatial position of the *v*cells within the cell cycle is initially determined from a real flow data set. From this information, each *v*cell is assigned a temporal position within the mean inter-mitotic time (μ_IMT_), allowing cell cycle events such as DNA replication and cell division to be stochastically predicted. After the *v*population has evolved for a given period, they may be compared with a further experimental data set to enable important simulation parameters, governing their evolution, to be optimised and constrained so that correlations between the respective data sets are maximised. Standard approaches to studying cell cycle involve statistical analysis of distributions either 1D involving nuclear content reporters [5] or 2D when further cell cycle molecular reporters are also included [1],[15]. Thus these are inherently ‘whole population’ measures and can only describe cell variability via global parameters such as the standard deviation from the mean. Whilst automated analytical approaches have been developed in order to reduce user subjectivity [16]–[18] the majority of flow analyses still involve user-defined gating of the as-measured data set to identify and segment a sub-population of cells. Subsequent mapping of this population onto 2D dot plots of fluorescence provides temporal snapshots (typically with a 24 hour sampling period) and further partitioning of cells to different compartments, G_1_, S, G_2_/M within normal and polypoid cycles (see Figure 1(a)). These apparent quantitative assessments become inaccurate and, to varying extent, subjective, as they are based either on user identification of the various components in the dot plot by fitting of Gaussian distributions, representing the G_1_, S, G_2_/M fractions, to the DNA content histogram [5]. The challenge of the current investigation is to adopt a computational approach, where the analytical and interpretive steps are implemented at the simulated biology stage and not on the raw data outputs. No new data is added in this approach and the computer simulations could be viewed as an elaborate form of data analysis. However, the methodology does deliver new insight on *process*, delivering a continuous simulation of the dynamic evolution of the cellular system between fixed sampling points. In this respect, it provides a physical validation when applying various hypotheses to interpret the experimental data. It also goes some way to visualising the variation between individual cells that gives rise to biological heterogeneity as the stochastic simulation delivers a report on population dynamics in which each and every cell can be tracked.

## Materials and Methods

### Experimental procedures

Experimental data is obtained using well-established bi-variate cytometric methods for study of the cell cycle: U-2 OS (ATCC HTB-96) cells were transfected with a G2M Cell Cycle Phase Marker (GE Healthcare, UK), yielding stable expression of a GFP-cyclin B1. This provides a green fluorescence signal the intensity of which correlates to position in the cell cycle with a minimum signal at G_0_ and a peak during the G_2_/M phase. [19]. The culture was maintained under G418 selection in McCoy's 5a medium supplemented with 10% foetal calf serum (FCS), 1mM glutamine, and antibiotics and incubated at 37°C in an atmosphere of 5% CO_2_ in air. To obtain fluorescence read-out of DNA content an anthraquinone derivative, DRAQ5™ (20 µM Biostatus Ltd., UK) was used [14]. This binds to DNA providing a fluorescence intensity that can be related to DNA content and thus it reports on cell cycle progression through the S phase to G_2_/M (>4N) or, in the presence of external perturbing agents, progression through polyploid states as the mitotic stage is by-passed [20] (see Figure 1(a)). To obtain a model system in which we can test the simulated cell population approach we have used a cell division by-pass agent: ICRF-193 [bis(2,6-dioxopiperazine)], a kind gift from Dr A.M. Creighton (ICRF, London, UK). This is a reversible catalytic inhibitor of topoisomerase II that blocks the ability of the enzyme to resolve interlinked DNA replication products [21]. The decatenation of chromosomal replication products is vital for the completing of segregation and hence normal division. ICRF-193, was prepared in DMSO at 2 mg/ml and used at a peak concentration of 2 µg/ml (equivalent to 7.2 µM).

To determine the cell population distribution of fluorescence intensity a FACScan flow cytometer was used (Becton Dickinson Inc., Cowley, UK) which was equipped with an air-cooled argon ion laser (with 488 nm output only). GFP-cyclin B1 data was collected using a 30 nm bandpass emission filter centred at 530 nm and the DRAQ5 signal with a 670 nm long pass filter. CELLQuest software (Becton Dickinson Immunocytometry Systems) was used for data acquisition. Flow cytometric analysis was used sample sets of 10,000 cells and the data presented represents the signal peak height. Typically, tracking of the population was carried out at 24 hour intervals.

### Virtual cell populace simulation

**Figure 2:**
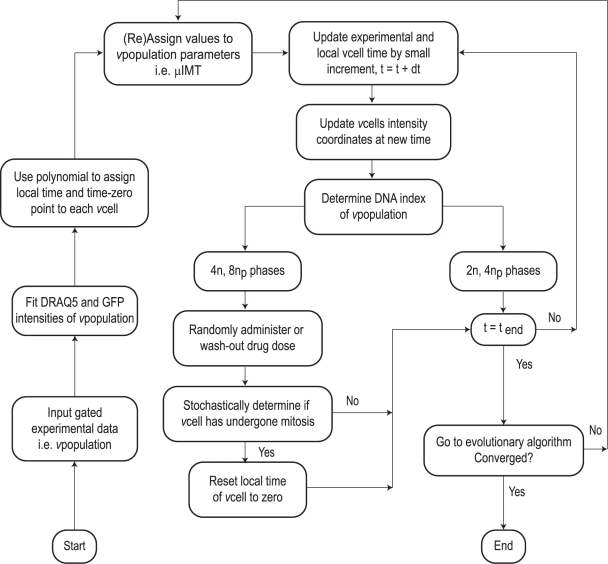
Flowchart indicating the main steps of the cell population model (CPM).

The computer simulation consists of two principal components; a cell population model (CPM) and an evolutionary algorithm - Differential Evolution (DE) [22]. The CPM generates a virtual population of cells (*v*cells), which is initialised using a flow data set. The *v*population is then evolved and compared to a subsequent flow data set. The CPM evolves each *v*cell and generates any cell cycle processes deemed relevant to explain the laboratory experiment. A DE algorithm is employed to optimise important ensemble parameters used in the CPM e.g. cell cycle time, enabling the *v*population to be evolved such that it maximises correlation with the data. A detailed description of the cell population simulation, complete with a full account of the various numerical algorithms and techniques used is given in Text S1. A brief outline of the main components of the cell population model is given in the following sections with reference to the simulation flowchart shown in Figure 2. All numerical algorithms have been written in the MATLAB environment (MathsWorks UK); fragments of pseudo-code for important aspects of the CPM are given in Text S1.

### Cell population model - initialisation

The *v*population is initialised by reference to a gated 2D flow cytometric data set composing of the cell cycle reporter cyclin B1 (GFP-cyclin B1) and DNA content determination (DRAQ5) (see Figures 1(b) and S1). The data is gated using a simple cell density cut-off technique, where a region is labelled active if its cell density is above a set threshold, (see Text S1). Cells within a contour encapsulating the gated fraction serve to initialise the *v*population position in the intensity space. The same gating procedure (and threshold value) is also applied to subsequent experimental data sets at later time points. More sophisticated gating techniques could be applied such as the expectation-maximisation algorithms presented by Boedigheimer *et al* [18]; however, this simple approach is adequate to establish the validity of our methodology.

**Figure 3:**
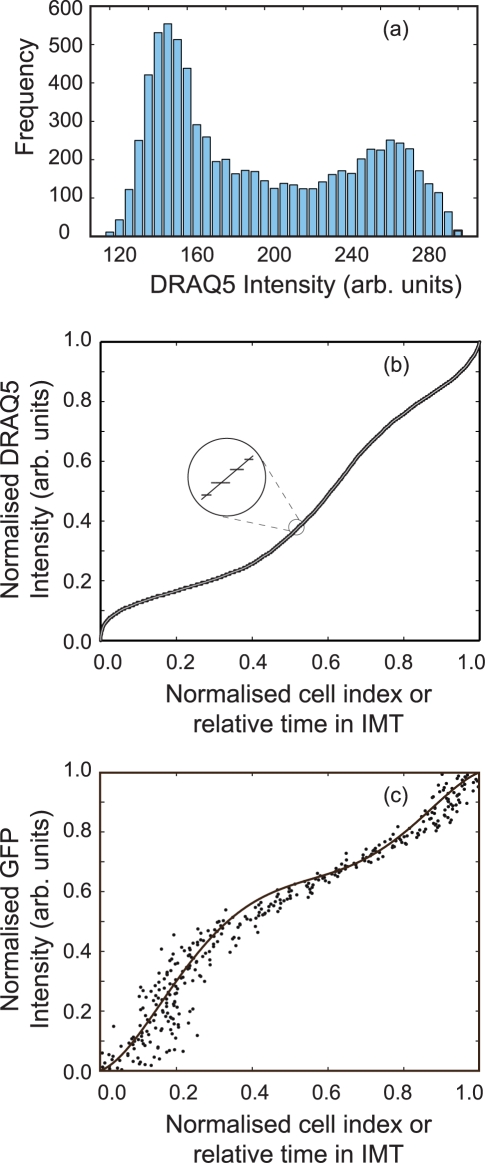
Comparison of traditional analyses of static flow cytometric data and to that used to develop dynamic data analysis. (a) Displays the fluorescent frequency histogram of the DRAQ5 intensity, plots (b) and (c) graphically illustrate how the median DRAQ5 and GFP cyclin-B1 fluorescence signals vary as a function of a normalised intermitotic time (light grey and blue lines respectively. *Insert*: plot (b) - magnification of the polynomial fit to the frequency histogram of the DRAQ5 intensity.

The gated data is now used to initialise the fluorescence intensities of the modelled cell population, which correspondingly inherits the biological variation seen in Figure 1(b) (each gated data point initialises one cell). The temporal position of each of the virtual cells within the cell cycle is unknown as the flow data (consisting only of only fluorescence intensities) contains no direct cell cycle time information. The time-based information, necessary to model the cell cycle dynamics, is extracted from the intensity signal of the biological markers obtained from the experimental data (see Text S1). The first approximation to assigning a time to each *v*cell is obtained by considering the DRAQ5 fluorescence intensity, the histogram of which is shown in Figure 3(a). In our approach, we use the DRAQ5, nuclear content indicator to position each *v*cell in the cell cycle making the following assumptions (i) the *v*cells are randomly distributed throughout their cycle and (ii) that their DRAQ5 signal is monotonically increasing through the cell cycle as the nuclear content is duplicated. This infers that the minimum and maximum DRAQ5 intensities correlate to the start and finish of the cell cycle and allows us to assign relative position in time to each *v*cell (see Text S1 and figure S2). The experimental dataset for DRAQ5 intensity is sorted into ascending order and fitted with a polynomial function (see Figure 3(b)). Because of the inherent digitisation produced by data binning, of the measured intensity, by a flow cytometer several cells will be recorded with the same DRAQ5 signal. The intensity sorting procedure assigns increasing sort indices to *v*cell sets with the same intensity (i.e. all cells within a given bin) the median index value is therefore used when implementing the polynomial fit (see inset in Figure 3(b)). Finally, the polynomial *x*-axis values are scaled to a range of zero to the inter-mitotic time (IMT - time between successive mitotic events), this gives an absolute time for each cell within its cycle. To relate the GFP signal to cell cycle time the intensity for each cell is plotted against the cell number index obtained from the DRAQ5 sort procedure, again fitted with a polynomial and scaled to give an *x*-axis running from 0 to the same IMT value (Figure 3(c)). The use of a stoichiometric nuclear content marker (such as DRAQ5) to estimate DNA content and hence cell cycle position is well established and both deterministic and stochastic models have been used previously to obtain continuous temporal descriptions [23]–[25]. Our approach differs from the previous studies in that through the creation of the virtual cell population we model at the level of single cells rather than using population level parameters.

The two fitting polynomials describe the evolution of the fluorescence intensities from cell birth to division as a function of time and are used to produce a median path through the cell cycle shown as the solid black line in the 2D GFP-DRAQ5 intensity plot displayed in Figure 1(b). It is obvious from the plot that many of the cells lie some distance from the median line this is due to natural variability in the measured signals caused by heterogeneity in reporter loading, noise, variation in collection efficiency etc. Each individual *v*cell is therefore assigned a cell cycle time by choosing a point on the 2D polynomial median line, that minimises the sum difference of the DRAQ5 and GFP intensity values (see Text S1 and figure S3). Therefore *v*cells at the same point within the cell cycle will display a heterogeneity in fluorescence signal value (corresponding to the width of the population plot in *x* and *y*-directions in Figure 1(b). Therefore, to calculate the time-dependent trajectory of each *v*cell through the 2D intensity space we update the DRAQ5 and GFP intensity values using the median line as time is incremented (see Text S1).

To summarise, the experimentally measured data is used to establish a virtual cell population with exactly the same heterogeneity in fluorescence signal as seen in the experiment. This *v*population is then evolved within a stochastic simulation allowing for variability in fluorescence intensity and IMT using a pair of polynomial functions that describe the cell cycle dependence of the signal, i.e. the absolute fluorescence is stochastic but the time evolution function is the same for all cells.

### Cell population model - evolution

Once initialised each member of the population has three discriminating properties corresponding to: (i) a time in the cell cycle, (ii) DRAQ5 fluorescence intensity and (iii) GFP-cyclin B1 levels. In order to mimic DNA synthesis and replication, a supplementary parameter, DRAQ5_DNA2_, is required; DRAQ5_DNA2_ details the DRAQ5 magnitude at which each *v*cell has doubled its DRAQ5 intensity (see Text S1 and figures S4 and S5). The CPM directly relates this to the point at which a real cell has doubled its DNA content, i.e. a phase transition to G2. The value of DRAQ5_DNA2_ is deduced by calculating the initial DRAQ5 intensity of each *v*cell at the start of the cell cycle (see Figure 1(b), black curve), which from the above is estimated at the effective intensity value just after a mitotic event, then assessing the time at which this initial intensity doubles using the polynomial functions shown in Figures 3(b) and (c).

Monitoring of the simulated DRAQ5 intensity then allows identification of cells that have multiplied their DNA content allowing placement of each into the following sub-groups: normal cycle – DNA index, DI = 2N (G1) or 4N (G2/M); polyploidy cycle - DI = 4N_p_ (G1_p_) or 8N_p_ (G2_p_/M_p_). Once *v*cells have entered the G2/M phase the probability of them entering the M phase and undergoing cell division is calculated. This is achieved using a simple stochastic decision process [13], where we define a cumulative Gaussian probability distribution which scales between 0 and 1, defined in terms of a mean inter-mitotic time, with an associated standard deviation, over the cell cycle time. Both these parameters are to be optimised via the evolutionary algorithm to best fit the second set of flow data. At each time step a random number, uniformly distributed in the interval [0 1] is generated and is compared with the cumulative probability distribution value at that time. If the random number is less than the probability distribution value calculated then mitosis is deemed to occur and the simulation generates two daughter cells at t = 0 in G1/S with the DRAQ5 and GFP-cyclin B1 associated with the parent cell. Otherwise, the cell remains in the G2/M phase for re-analysis at the following time step, which will increase both of its intensity coordinates resulting in a higher probability of mitosis (the cumulative Gaussian distribution tends to 1 with increasing time). The implementation of this mitotic variability produces further heterogeneity in the IMT of the individual *v*cells.

The cell population model is defined by a set of parameters specific to the flow cytometry experiment conducted. Optimisation of the fit between simulation and experiment is dependent upon selection and minimisation of the population variables, in our case: the mean inter-mitotic time, its standard deviation and a parameter detailing the presence of a drug in the *v*population. There are several different methods, which could be used to determine the best fit to the experimental data; we choose to use a differential evolutionary technique to optimise these cell cycle parameters. The quality of fit associated with a set of CPM parameters is determined by calculation of the ratio of evolved *v*cells to that measured experimentally within a numerically deduced gated region. This simple maximisation strategy, works well for both therapeutically (un)perturbed systems, although newer versions of the CPM will explore more sophisticated 2D cross correlative algorithms to infer fitness. Convergence of the differential evolution algorithm is determined true when the quality of fit varies by less than 1% over five subsequent generations (see Text S1).

## Results

### Cell population simulation

**Figure 4:**
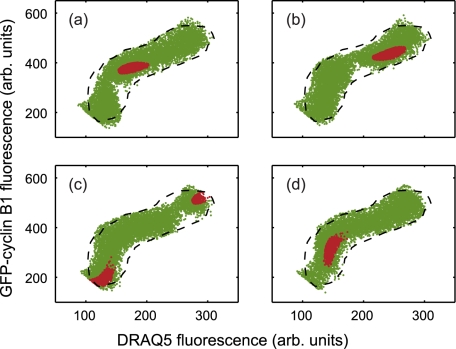
Model simulation to track a virtual sub-population (red markers) of *v*cells through the cell cycle at (a) 6 hours (b) 12 hours (c) 18 hours and (d) 24 hours. Note: the overall *v*cell population has a mean intermitotic time of 22 hours and an associated standard deviation of 6 hours.

To illustrate the evolution of the *v*cell population, we generate a series of snap-shots derived at different temporal intervals (Figure 4 - green population) demonstrating the simulated intensity dot plot at 6, 12, 18 and 24 hours respectively after initialisation by an experimental data set. Here, the *v*cell population has a mean IMT of 22 hours and an associated standard deviation of 6 hours; a small subpopulation of *v*cells can be depicted (red dots) also a contour (dashed black line) is displayed, indicating the extent of the gated experimental data set at initialisation. Given that the cells in these experiments are randomly distributed within their cycle and a statistically relevant data set is sampled the acquired plots appear identical for a control sample with an unperturbed biology. The advantage of the simulated population approach is therefore evident in Figure 4, as a discrete sub-set of cells is identified and its dynamics tracked over a period of time. Despite using a single experimental sample information is obtained across the whole of the cell cycle due to the assumption of random temporal distribution. The fundamental insight gained here is the adoption of a simulated cell approach and subsequently the visualisation of the temporal dimension encoded in the fluorescence intensity distributions.

### Cell cycle perturbation and cell cycle rerouting to polyploidy

To test the ability of the simulation to capture more complex dynamics associated with aberrant cell cycle progression and variance of response across sub-populations a cell cycle perturbation experiment was undertaken using a mitotic by-pass agent ICRF-193. Cells treated with this agent progress through multiple replication cycles without undergoing mitosis, therefore doubling DNA content [21]. This leads to an evolving polyploid population that is identified using the nuclear dye, DRAQ5 to obtain an optical read-out of DNA content. Perturbation of the cell cycle and rerouting of cells in this manner provides a system in which the population dynamics of diverted sub-groups within the normal and polyploidy cycles can be analysed. The challenge for the cell population simulation is to track the inter-related pharmacodynamics, taking full account of the detailed evolution of the accompanying fluorescence data.

### Experimental outputs

**Figure 5:**
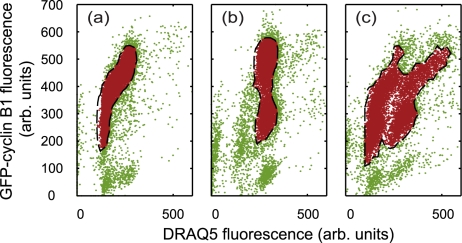
Endocycle routing with ICRF-193 (a), (b) and (c) indicate experimental data (green markers), gated sub-set (red markers) and a contour of the gated data (dashed black line) at 0, 24 and 42 hours respectively.

A block and chase experiment was conducted in which cells were continuously treated with ICRF-193 for 24 hours (Figure 5(a–c)). A 2D dot plot of the cell cycle (GFP-cyclin B1) and nuclear content (DRAQ5) reporters at the 24 hour time point shows a sub-population of cells with low GFP-cyclin B1 expression and a DNA index of 4N i.e. polyploid cycle cells in the G1/S phase (Figure 5(b)). Compared to control conditions, where all cells were engaged in the normal cell cycle. Following the 24 hour drug treatment with ICRF-193, wash-out allows cells to further cycle unperturbed under normal conditions for a further 18 hours (including cell division). ICRF-193 is a reversible topoisomerase II blocker and so removal of this agent enabled the sub-population of cells within G2/M of the normal cycle to be routed back into normal cycle (i.e. to G1/S). Hence, the 42 hour data shows two distinct population groups describing cells within the normal and polyploid cycle (Figure 5(c)).

### Model outputs

**Figure 6:**
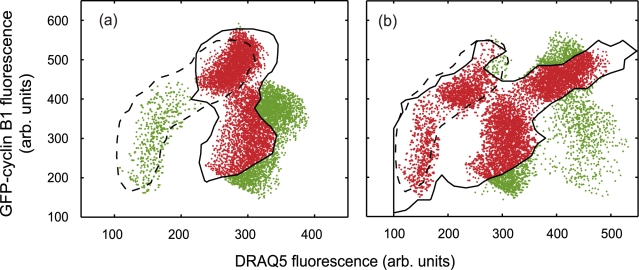
Simulated *v*population at (a) 24 and (b) 42 hours respectively. The red shaded areas are virtual cells that lie within the respective experimental gated contours shown in Figures 6(b) and (c), the green shaded points are simulated data lying outside of these contours. Also indicated is the gated, time zero experimental data set contour (black dashed line).

To include the effect of the ICRF-193 in the CPM we include a further optimisation parameter *N_bp_*, which describes the fraction of *v*cells that have doubled their DNA content (G2/M phase) but have bypassed mitosis. These are selected stochastically and inhibited from undergoing cell division when under drug ‘dosing’ conditions. This assignment is undertaken at each time step, until the required percentage of *v*cells in the population have by-passed mitosis. In the drug ‘wash-out’ conditions, the reduction in *N_bp_* is modelled with a half-life, *t_1/2_*, corresponding to the temporal persistence of the drug-induced perturbation. Thus depending on drug administration or wash-out the CPM has three optimisation variables to be minimised through the evolutionary methods described previously. The comparison of real (red population displayed in Figure 5(b)) and *v*cell populations for selection of the variable parameter values is made 24 hours after initialisation. The optimised *v*cell population together with the real data contour is shown in Figure 6(a) together with a contour illustrating the position of the initial data set. The simulation clearly captures the key features of the population evolution and given the stochastic nature of both real and virtual cell populations they are well correlated. At this point, following 24 hours of continuous drug treatment, there are large fractions of 4n cells in the normal and 4n_p_ polyploid phases as well as a sub-population of polyploid cells progressing to 8n_p_ phase. A small population sub-set (located within the black dashed contour in Figure 6(a)) represents the *v*cells yet to be influenced by the drug. As mentioned above, at each discrete time throughout the simulation the *v*cells are stochastically tested to see if they have been drug treated, hence, a finite time must elapse before all *v*cells can be influenced by the action of the drug.

The *v*cell dynamical parameters corresponding to the fits shown in Figure 6 are indicated in Table 1. In the presence of the drug the simulation indicates a mean inter-mitotic time of 36 hours with a standard deviation of 4 hours. In comparison, the IMT value from fitting to a control set of data is 22±4 hours. Multiple runs (1,000 simulations of the experimental data) of the model indicate that the variation in the tabulated values, due to stochastic variation and evolutionary selection, is less than 5%. The prediction of an extended IMT within drug treated cells is in agreement with previous studies on the effects of ICRF-193, showing delays in progression to the mitotic phase plus extension in the duration of mitosis once initiated. Although, the CPM cannot elucidate on the persistence of individual phase duration it does accurately estimate their combined effect.

**Table 1:** Optimised parameter values of the *v*cell population.

Parameter	μIMT (hours)	σIMT (hours)	*N_bp_* (%)	*t_1/2_* (hours)
**Drug treated (0–24 hrs)**	36	6	99	-
**Wash-out (24–42 hrs)**	22	7	-	3

During the chase phase of the assay subsequent to drug wash-out, the simulation evolves from 24 to 42 hours in a similar manner to that above, with the difference that the *N_bp_* parameter is indirectly optimised using a half-life to describe its temporal decay; i.e. the fraction of *v*cells that retain drug-induced division-bypass is 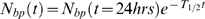 where 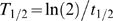 and *t* is the time since wash out. The intensity coordinates of the *v*population at 42 hours after ICRF-193 washout are displayed in Figure 6(b). The simulation has captured the important features of both the normal and polyploid cycle dynamics. That is, there is a significant sub-population of *v*cells in each of the four DNA indexed phases. For the 2D fit shown in Figure 6(b) the simulation uses a mean inter-mitotic time of 22±7 hours respectively. This agrees remarkably well with that measured through microscopic techniques for an unperturbed real populace. Furthermore, the simulation gives an insight to the temporal persistence of the drug on the virtual population, indicating that a significant sub-population retain or are committed to the division bypass over the course of a few hours following wash-out. The fact that an effective continuum of intensities straddling the 4n_p_ and 8n_p_ phases in both real and virtual populations is evident reinforces the simulation result which highlighting of temporal persistence of ICRF-193 post washout. The evolution and perturbation of the *v*cell population is shown in Video S1.

**Figure 7:**
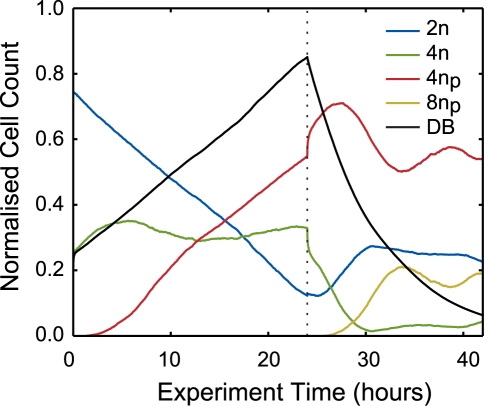
The normalised virtual cell count throughout the time-course of the experiment. The blue, green, red and yellow curves indicate the fraction of virtual cells in the 2n, 4n, 4n_p_ and 8n_p_ phases of the cell cycle respectively. In addition, the fraction of virtual cells drug blocked throughout the simulated experiment is indicated by the black curve.

The continuous population dynamics provided by the simulation are shown in Figure 7. At the initialisation point (t = 0 hours) we see that a significant fraction of *v*cells are present in the 2n phase compared to that in the 4n phase (blue and green curves respectively), ∼4∶1 ratio. Over the first 24 hours, the drug perturbation re-routes cells from the normal into the polyploid cycle. Thus, the 4n population is stable as equal numbers of move in and out of it producing linearly decreasing 2n and linearly increasing 4n_p_ sub-populations. The percentage of mitotic-bypass cells therefore increases over time, but due to dynamical constraints and the optimised mean inter-mitotic time of 36 hours, this does not reach 100% (maximum of ∼85%) before washout. The vertical dotted line in Figure 7 indicates the initiation of the washout phase of the simulated experiment. Following drug washout at 24 hours the fraction of mitotic-bypass *v*cells decreases exponentially with an optimised half-life of 3 hours, thus it takes the full 18 hours following drug removal to achieve something near to normality. This same dynamic inevitably affects the re-creation of a 2n population. This gives an insight to the temporal persistence of drug on the *v*population indicating that a sub-population retains the bypass commitment for a few hours post washout.

## Discussion

The use of stochastic computing approaches plus evolutionary algorithms to evolve a simulated cell population provides a new approach to the analysis of multi-variate data sets obtained by flow cytometry. In using this simulated biology process to analyse cell cycle perturbation we have obtained detailed information cell cycle time and the detailed dynamics of cell division and proliferation. Furthermore, we have shown that a subpopulation or cohort can be defined and tracked throughout the time course of the experiment without the need for further molecular markers, this can be essentially viewed as an *in silico* representation of the pulse chase experimental methods such as those incorporating two-parameter flow cytometry analysis: with DNA content and BrdUrd [26]. When applying the technique to drug-treated populations the pharmacodynamic indicators can be tracked and sub-populations within normal and polyploid cycles differentiated. Further, the temporal continuity inherent in the computational assessment also highlights details un-resolvable in the experimental sampling, such as cell cycle traverse (inter-mitotic time variation), cell cycle delays (persistence of drug-induced effects) and has also identified the occurrence and location of cell cycle restriction points, which with additional molecular mapping can be further defined [27]. Also, the simulated experiment permits individual in addition to (sub)population cell tracking allowing single cell lineage tracking and the ensuing generational patterns and relationships to be continually analysed. This is a systems approach to whole tumour population evolution leading to lineages, in contrary to tracking individual lineages and extracting a global population response [28]. We envisage that this approach would be much more easily applied to a screening approach appropriate for sampling tumours both *in vitro* and *in vivo*.

In this initial implementation of the technique, we use a cell cycle marker that reports on relative cycle time and a nuclear marker which allows us to discriminate between normal and polyploid cell populations, therefore no further information of the intricate details of the cell cycle (apart from mean IMT distribution) can be deduced. In this respect, the simulated cell methodology provides a framework, describing the relationships between cells within a population, at a system level i.e. in the context of progression through a unitary cycle with associated genetic replication and cell division. Importantly this structure can enhance existing approaches by linking detailed molecular level models of cellular evolution through specific cell cycle phases [26],[27],[29] to cell heterogeneity and its influence on population level dynamics.

We have adopted an approach of minimised complexity in order to clearly demonstrate the concept without the obfuscations of detailed algorithm structures and data filtering. A simple dot density cut-off filter is applied to gate the data, the number of variable parameters within the genetic algorithms is reduced to a minimum of three and goodness of fit assessed by a straightforward maximisation of simulated cells within an experimental data contour. Whilst future work will explore the potential of more sophisticated computational techniques, the simple conceptual base presented here already provides automated, objective data analysis that encapsulates the fundamental biology and delivers statistically robust results. Given the stochastic nature of the simulation it could be argued that a statistical approach should be maintained and increased simulation runs be used to acquire added certainty rather than increased model complexity. The large data sets collected in flow cytometry and the stochastic variation associated with biological systems naturally lead to statistical analysis techniques for data interpretation [23]–[25]. These have proven to be powerful tools in cell biology, however when focussing on individual cell behaviour and heterogeneity expressed at the single cell level the integrative measures of statistics are limiting. The development of a simulated biology, twinned to a real cell population, by fitting experimental data sets, maintains the statistical relevance and provides discrimination via individual cell recognition. The creation of *in-silico* cells brings the potential for interpolation and extrapolation thus a *continuous* temporal report of complex population dynamics can be produced from discrete measurements and cellular behaviour predicted beyond the limited time frame imposed by experiment and environment. The temporal continuity inherent in the computational assessment also highlights details of the pharmacodynamics, un-resolvable in the experimental sampling, such as inter-mitotic time variation and persistence of drug-induced effects. Perhaps the most beneficial aspect of the simulated cell approach is its ability to provide direct knowledge of biological state allowing a computational systems approach to inform the biology. This contrasts with traditional flow analysis, which provides information that is primary in relation to data but secondary in relation to cells; i.e. a choice can be made between direct data analysis with interpretation to translate to cell behaviour or direct read-out of cellular information from a data-directed simulation. By ensuring interoperability of the modelling algorithm with experimental cytometry outputs, the simulation provides emergent features of the cell cycle and the functional operation of molecular restrictions and checkpoints; providing further the foundation for considering the evolving asymmetric and symmetric patterns of a dynamic cellular system.

## Supporting Information

Text S1 Information (detailed information, figures and movies) will be given a leading ‘S’ to designate that they are present within Supplementary Information.
(0.22 MB DOC)

Figure S1 Flow cytometry data set indicating the DRAQ5 and GFP-cyclin B1 fluorescence intensities of a measured cell population. This plot highlights the raw data (grey) and the subsequent gated fraction (red); also, the three principle sub-fractions present within the raw data are numerically labelled.
(1.78 MB EPS)

Figure S2 Fluorescence intensity plot of the normalised vpopulation; vcells highlighted in red refer to those with the lowest normalised DRAQ5 values.
(2.79 MB EPS)

Figure S3 Plot indicating the curve v's over the cell cycle interval [0 IMT], and the vpopulation, 3 members of which have been highlighted (yellow markers) with DRAQ5 and GFP intensity differences (red arrows) corresponding to equations [S2.3.2]. *p_DRAQ_*(*t*)*p_GFP_*(*t*)
(2.59 MB EPS)

Figure S4 Figure indicating location of the roots (yellow markers) between the median intensity line and linear functions (that enclosed by red ellipses) describing evolution of DRAQ5 and GFP intensity coordinates as a function of time.
(2.61 MB EPS)

Figure S5 Intensity plot highlighting the properties of three randomly chosen cells. These properties include: their vcells intensity coordinates at both t and t marked in yellow and red respectively and their corresponding intensity trajectory through their cell-cycle (solid black lines). *v*_E = 0t = 0_
(3.00 MB EPS)

Video S1 VPopulation simulation
(2.61 MB WMV)
