# Supplementary material for: Flow-Based Cytometric Analysis of Cell Cycle via Simulated Cell Populations
Source: PLoS Comput Biol. 2010 Apr 15;6(4):e1000741. doi: 10.1371/journal.pcbi.1000741 (PMC2855319; doi:10.1371/journal.pcbi.1000741)
Supplement: Text S1 — Information (detailed information, figures and movies) will be given a leading ‘S’ to designate that they are present within Supplementary Information. (0.22 MB DOC) [file pcbi.1000741.s001.doc]

**Supplementary Information**

**Flow-based cytometric analysis of cell cycle via simulated cell populations**

**S0 Cell Population Model (CPM) Overview**

The CPM generates a *virtual* population of cells (*v*population, *v_p_* or *v*cells), the properties of which are attained from a biparameter flow cytometry data set, *E*_0_, at time, *t*_0_. In this particular case, experimental measurements are obtained for 10,000 cells in the form of two fluorescence readouts which correspond to two independent reporters that reflect cell cycle position; the first the DNA content and the second to the GFP-Cyclin B1 content or expression of a cell respectively (Details of these cellular reporters are summarized in the Materials and Methods section of the manuscript). The status of this two dimensional (2D) distribution was then monitored from time series flow data with and with out a drug perturbation (e.g. the topoisomerase II inhibitor ICRF-193) represented at *t*_0_, *t*_1_, *t*_2……_ .

The two cellular reporters are used at *t*_0_, as the initial coordinates in the 2D intensity space, by the CPM to relatively position each member (ie 10,000 cells) of the populace within this domain. The intention is then to use the CPM to evolve these individual coordinates from *t*_0_ to a later time in the time series (eg *t*_1_), where a second flow cytometry data set, *E*_1_, is used, to determine and ‘make a judgment’ of the correlation of the updated intensity coordinates of the *v*population to that of *E*_1_. To maximize correlations between these data sets *E*_0_ and *E*_1_ a differential evolution (DE) algorithm is employed to optimize the important ensemble parameters, such as intermitotic time (IMT).

To construct a framework by which temporal evolution of the *v*population maybe achieve several initialization stages on the set *E*_0_ are required:

1. The experimental data *E*_0_ requires a sensible gating strategy to ensure that we consider only the viable intact fraction, *v_f_*, of reporting cells, e.g. we do not include debris and dead cells and importantly we remove the non-reporting GFP-cyclin-B1 fraction. The intensity properties of viable fraction initialise the properties of the *v*population at *t*_0_ .i.e. (*v_p_*(*t*_0_) ≡ *v_f_* ⊂ *E*_0_).
2. The intrinsic properties of *v_p_* are used to determine numerical strategies to enable cell cycle progression or evolution in the intensity space for each *v*cell member as a function of time. Also, these allow assignment of additional components to every member of the *v*population; these components describe initial temporal position within the cell cycle at *t*_0_, taking into account heterogeneity across the populace compounded by the variation of intensity of each component.
3. Using information gained from point 2 above, each member of *v_p_* are assigned two further attributes. The first describes the intensity coordinates of the *v*cell following mitosis, which can be thought of as its position in intensity space at its local time zero point. The second details the magnitude of the DRAQ5 (DNA content) intensity labeling entrance of the *v*cell into the G2/M phase from the G1/S phase of the cell cycle. This magnitude is determined by simply doubling the value of the DRAQ5 signal at its local time zero point.

Thus, section S1, details the numerical strategies and postulates employed to accomplish the above points. Following this in S2, we detail how the information gathered in S1 can be used to initialize *v*cell parameters required for their evolution in the 2D intensity domain, discussed in section S3 and S4. We also, indicate the optimization strategy employed (section S5) when fitting the evolved vpopulation to a second flow cytometry data set at time t_1_.

All numerical algorithms developed during the course of this work have been written in the MATLAB environment; fragments of pseudo-code for some important aspects of the CPM are given in S7.

**S1 Gating**

Gating of the data is required to infer a reporting/viable fraction of cells, *v_f_*, from within the measured experimental data set *E*_0_, i.e. *v_f_* ⊂ *E*_0_. Gating of the experimental data set is important as there will exist a fraction of non-reporting cells that will be detected by the flow cytometer (e.g. with a 1D DNA content only attributes); further these may also be derived from dead cells or cells that have been damaged or stressed in some way. However, we show that the intensity profiles of this non-viable and reporting subset does not adhere to that exhibited by the general population. The grey and red markers of Figure S1, indicate the experimentally acquired data set, *E*_0_. It is quite evident that there are three main populations of cells; (1) low DRAQ5 (< 50 intensity units) with a range of GFP signal – this is cellular debris and dead cells (2) low GFP (< 100 intensity units) with a normal range of DRAQ5 signal – these are viable GFP-cyclin B1 empty (non-transfected) cells that only have access to the DRAQ5 reporter and (3) the predominant dual labeled viable fraction, which the gating procedure seeks to locate. In this case, *v_f_* is deduced by a simple density cut-off technique. Here the 2D intensity space, encompassing both GFP-cyclin B1 and DRAQ5 signals is binned into a square grid; each square element has side of 15 intensity units. Each intensity element is then sequentially visited and the number of data points,$n_{d}$, within it deduced. If $n_{d}$ is above a set threshold criterion (in this case 25 cells (data points)) the elemental area is labelled active. The set of all active elemental areas, A, are connected to form a closed *N*-sided polygon, defining the contour (dashed line) see manuscript Figure 1(b). The last step of the gating procedure calculates the intersection of sets A and E to give the set *v_f_, (v_f_* = A ∩ E) of data points that lie on or within the contour.

The set *v_f_,* initializes the intensity coordinates of the virtual cell population at time*, t_0_*, i.e. *v_p_*(*t*_0_) ≡ *v_f_* ⊂ *E*_0_). Thus, when either the DRAQ5 or GFP-cyclin B1 signal is subsequently referred to, we mean this to be the viable fraction of these signals deduced from gating. The distribution of the gated intensity coordinates of the *v*population members are indicated in red (Figure S1).

This simple gating procedure is applied to all time-series experimental data sets (e.g. see figures 5(a) – (c) in the manuscript) to allow objective segmentation of data at all time points. However, due to the dilution, in intensity space, of the experimental data at later times; a fairly low threshold criterion was employed causing the contouring algorithm to include regions of intensity space seemingly devoid of cells. Future versions of the CPM will implement more sophisticated segmentation methods.

**S2 Initialization of time in cell cycle index**

The second initialization section of the CPM develops numerical strategies to evolve the intensity coordinates of the *v*population as a function of time. The fluorescent intensity signals of the virtual cells convey relative position within a cell cycle (described in this case by two parameters); they contain no direct temporal information. In order to achieve a chronological assignment, the CPM assumes the following criteria to be true: (i) the *v*cells are randomly distributed throughout the IMT interval and (ii) that the DRAQ5 signal is monotonically increasing throughout the cell cycle, therefore the minimum and maximum intensities of this signal correlates and therefore defines the start and end of the cell cycle. These two important criteria, present a temporal framework in which each *v*cell can be assigned a third component: an initial time coinciding with their relative position in intensity space with respect to other members of the populace. Furthermore, these linked postulates provide a means to update both their DRAQ5 and GFP fluorescence intensity coordinates as a function of time, allowing evolution of the populace through interphase up to mitosis and subsequent evolution of progeny (daughter) *v*cells through their corresponding cell cycle.

***S2.1 Intensity signal transformation***

Before temporal assignment, it is numerically convenient to transform the DRAQ5 and GFP components of the *v*cell populace to the [0 1] × [0 1] unit square in the plane ^2^ with corners (0,0), (1,0), (0,1), and (1,1). This transformation is easily achieved by normalization of the intensity coordinates through equation S1.2.1.

$\xi_{i}^{n}=\frac{\xi_{i}-\min(\xi)}{\max(\xi)-\min(\xi)}$ *i* ⊂ *v_p_* and *ξ* = DRAQ5 and GFP [S1.2.1]

where $\max(\xi)$ and $\min(\xi)$ refer to the maximum and minimum values of the respective intensity signals and$\xi_{i} or$and $\xi_{i}^{n}$refer to the original and normalized values of intensity of the *i*^th^ *v*cell. The values of $\max(\xi)$ and $\min(\xi)$ for each intensity signal are stored to permit renormalization to the experimentally measured intensity domain from that of the unit square. Figure S2 displays the normalised intensity coordinates of the virtual populace. Also highlighted (red markers) on this plot are the *v*cells with the lowest DRAQ5 signal, the significance of their 2D location is discussed below.

***S2.2 vpopulation Dynamics***

In order to initialize a relative position of each *v*cell within the cell-cycle we first make use of the postulates (i) and (ii); numerically sorting the normalized DRAQ5 component of the populace in ascending order, we transform the data once more to form a monotonically increasing stepped-function, akin to an empirical cumulative distribution [1], which for convenience we call $D_{CF}^{n}$. The step features present in $D_{CF}^{n}$, arise from both the finite experimental resolution when measuring the intensity signal and intrinsic cell heterogeneity; $D_{CF}^{n}$ is plotted as a function of *v*cell index in figure 3(b) of the manuscript. The significance of this curve is that if we believe postulates (i) and (ii) and that cells double their DNA content in a uniform manner if unperturbed then $D_{CF}^{n}$describes how the DRAQ5 intensity component of a *v*cell will evolve through its respective cell cycle. Furthermore, adherence to these criteria places temporal boundaries on the *v*population, i.e. minima and maxima of $D_{CF}^{n}$ refer to the start (local cell time = zero) and finish (or total time or intermitotic time) of the cell cycle. Thus by fitting, $D_{CF}^{n}$via a suitable polynomial function we reveal a means to dynamically update the DRAQ5 coordinate of every member of the *v*population as a function of time.

To ease fitting of the polynomial to $D_{CF}^{n}$we reduce $D_{CF}^{n}$so that at each discrete intensity bin, $\delta I$, where the *j*th bin is given by $\delta I_{j}=\sum_{i=1}^{j} \frac{1}{N_{cells}}$, the range of *v*cells with corresponding intensity, $\left.vcell_{j}\right|_{\delta I_{j}}$, is replaced by a single integer value, $d_{CF}^{n}$, closest to the median of the spread, i.e. $d_{CF}^{n}=round\left[median\left(ran \left(\left.vcell_{j}\right|_{\delta I_{j}}\right)\right)\right]$, where $d_{CF}^{n}$⊂ $D_{CF}^{n}$.

We next numerically fit $d_{CF}^{n}$ with the function, $p_{DRAQ5}(t)=a_{n}t^{n}+a_{n-1}t^{n-1}+\ldots+a_{2}t^{2}+a_{1}t+a_{0}$ to reveal a continuum curve describing how the DNA content of a *v*cell will vary over time. The black data points display the $D_{CF}^{n}$ and the solid blue line represents a polynomial in time fitted to$d_{CF}^{n}$. The inset in figure 3(b), displays an enlargement of $D_{CF}^{n}$revealing its stepped nature and the continuum of the $p_{DRAQ5}(t)$curve; the *x*-axis of this figure is the normalized *v*cell sorted index number of$D_{CF}^{n}$, i.e. the number of *v*cells divided by *N*_cells_, so that we occupy the unit interval due to the postulates detailed above, this unit interval can now be thought of as the intermitotic time.

This procedure cannot be strictly applied in the same manner to reveal a second polynomial function that represents the GFP component evolution because unlike the DRAQ5 intensity the properties of this intensity distribution, in general, need not be monotonically increasing throughout the cell cycle. We therefore use the normalized GFP coordinates of the *v*cells that are members of the set $d_{CF}^{n}$, which we label $g^{n}$. We can then similarly fit a second polynomial, $p_{GFP}(t)$, to this reduced data set to provide a mechanism to evolve the GFP intensity coordinate of each *v*cell as a function of time. This distribution along with a curve depicting a polynomial fit to the data is displayed in figure 3(c) of the manuscript; the black markers and solid blue line respectively. Figure 3(c), is clearly illustrates the variability associated to the distribution$g^{n}$, which complicates the process by-which an initial cell cycle time is assigned to each member of the *v*population. If this variability were not present, an initial time could be deduced directly from $D_{CF}^{n}$ (see figure 3(b)), i.e. simply assign a time according to sorted DRAQ5 position over the interval [0 1], values of which can be multiplied by an appropriate intermitotic time to reflect ‘real’ cell cycle dynamics (N.B. the intermitotic time is a CPM optimization variable). The actual method for initial cell cycle assignment is detailed in the following sub-section; this sub-section has used two important postulates about the experimental data set obtained to deduce two polynomial functions that describe how both the DRAQ5 and GFP-cyclin B1 signal may vary in magnitude over a *v*cells cell-cycle.

***S2.3 Time during cell cycle allocation***

To assign an initial cell-cycle to time to the virtual population we make use of the two previous deduced polynomial functions $p_{DRAQ5}(t)$ and $p_{GFP}(t)$ and their derivatives to implement a 2D Newton-Rhapson minimisation routine [3]. The function to be minimised is:

$f(t_{0})=f_{DRAQ5}(t_{0})+f_{GFP}(t_{0})$ [S2.3.1]

where

$\begin{matrix} f_{DRAQ5}(t_{0})=p_{DRAQ}(t_{0})-g_{DRAQ} \\ \\ f_{GFP}(t_{0})=p_{GFP}(t_{0})-g_{GFP} \end{matrix}$ [S2.3.2]

here, $p_{DRAQ}(t), p_{GFP}(t)$ are vectors of length *N*_cells_ (i.e. describing the *N*_cells_ members of the virtual population) referring to the polynomial values at time *t*_0_ and $g_{DRAQ}, g_{GFP}$refer to the magnitudes of the DRAQ5 and GFP signals of the *v*population respectively. The initial time, *t*_0_, allocated to the each member populace are generated from *N*_cells_ random numbers uniformly distributed over the unit interval [0 1]. These initial temporal values are then iteratively refined through the expression:

$t_{1}=t_{0}+J(t_{0})$ [S2.3.3]

where

[S2.3.4]

is the Jacobian, deduced from the ratio of equation [S2.3.1] and its derivative, which gives a numerical update to the initial temporal values *t*_0_ assigned to the virtual populace. This iterative procedure if repeated until the magnitude of the L^2^-norm of the Jacobian falls below 10^-8^. Members of the minimised set of temporal values at the start of the virtual experiment, *t*_E=0_, are then assigned to the corresponding *v*cells.

In figure S3, we show the ‘median line’ of the populace in intensity space that is deduced plotting the DRAQ5 and GFP polynomials against one another. Due to the postulates detailed in the previous sub-section, the intensity coordinates of each *v*cell will follow a similar shaped trajectory, only its relative position in intensity space will be shifted. The red arrows of three randomly chosen *v*cells in figure S3 indicate the area to which the shift is limited. Thus, the minimization procedure effectively re-positions or calculates the root of the median line with the DRAQ5 and GFP coordinates of the each *v*cell in order to assign an initial time within the cell cycle. Furthermore, the allocation of a randomly assigned from the intermitotic time interval to initialize the Newton-Rhapson minimisation routine maintains the relative intensity signal broadening.

***S 2.4 Polynomial fits***

In both cases, the CPM works to find the lowest order polynomial describing the variation of the two intensities; this is mediated by monitoring and minimizing the L^2^-norm [1] against polynomial order, i.e. if the L^2^-norm is decreased by less than 5% going from order *n* to *n*+1, the *n*th order is retained. Figure S3 displays the solid black line is the curve resulting from the combination of the two fitting polynomials. It is evident from the curve that there are two linear regions at its extremities; these are present to inhibit numerical infinities that are manifest in the polynomial functions near the two temporal boundaries, a consequence of the sorting procedure. To avoid these numerical deficiencies linear functions are employed to describe the evolution of the DRAQ5 and GFP intensity coordinates of the *v*cells for cell cycle times less than 5% and greater than 95% of the mean intermitotic time. The two roots for each curve describing polynomial intersection with these linear functions were determined via a Newton-Rhapson iteration scheme [3]; the locations of the roots are indicated by the red circles in figure S4. The location of these two roots numerically defines the bounded cell cycle system. The gradients of the linear functions were optimized to allow a continuous path across the roots to avoid discontinuities in both DRAQ5 and GFP signals.

**S3 Cell population model – *v*cell evolution**

Once initialized, each member of the population has three discriminating properties corresponding to, *t*_E=0_ (i) DRAQ5, (ii) GFP-cyclin B1 fluorescence intensities (or coordinates) and (iii) a cell cycle time index at the start of the experiment. In order to mimic DNA synthesis and subsequent path through mitosis, two further parameters for each *v*cell are required. These are the DRAQ5 and GFP coordinates at the start of the cell cycle subsequent to a mitotic event, i.e. this temporal position is the ‘local’ (to each *v*cell) start or time zero of the individual cell cycle of the populace, *t*_t=0_.

$\begin{matrix} \xi_{i}(t_{t=0})=p_{\xi_{i}}(t=0)-\Delta\xi_{i} \\ \\ \Delta\xi_{i}=\xi_{i}(t=0)-p_{\xi_{i}}(t_{E=0}) \end{matrix}$ *i* ⊂ *v_p_* and *ξ* = DRAQ5 and GFP [S3.1]

The magnitude of both the DRAQ5 and GFP intensity at *t*_t=0_ are calculated via expression S3.1, this is the summation of the individual *v*cell intensity at time zero and that at the start of the experiment, *t*_E=0_ minus the initialized magnitude at *t*_E=0_ deduced experimentally. Figure S5, indicates the *v*population together with the intensity coordinates of three randomly chosen members of *v_p_*, marked in yellow also shown is the corresponding *t*_t=0_ intensity coordinates of these *v*cells (marker in red). Thus, after the procedure each *v*cell has five distinguishing features, DRAQ5 and GFP intensities at *t*_E=0_, *t*_t=0_ and a time *t*_t=0_ in cell cycle. From the values DRAQ5(*t*_t=0_) we can deduce one further quantity, DRAQ5_DNA2_ = 2×DRAQ5(*t*_t=0_), that is the magnitude of DRAQ5 intensity after DNA synthesis through S-phase each individual *v*cell has to surpass for an effect transition from G1/S phase to the G2/M phase of the cell cycle. The CPM directly identifies this point to the point at which a real cell has doubled its DNA content. Monitoring of the simulated DRAQ5 intensity then allows identification of *v*cells that have multiplied their DNA content allowing placement of each into the following sub-groups: normal cycle – DNA index, DI = 2N (G1) or 4N (G2/M); polyploidy cycle - DI = 4N_p_ (G1_p_) or 8N_p_ (G2_p_/M_p_). Once a *v*cell has passed the DRAQ5_DNA2_ point and entered the G2/M phase the CPM stochastically samples the individual to test for the mitotic event; this stochastic decision process is detailed in the following sub-section and a flow diagram detailing every step of the CPM is displayed in figure 2 of the manuscript. Once the aforementioned parameters have been defined for each member of the *v*population, the CPM is now in a position to evolve this set to a new temporal point, i.e. $t=t_{0}+\delta t$. Typically, we employ a time increment, *δt*, of ~ 15 minutes to update the *v*population from one temporal location to another. The intensity coordinates of each *v*cell is then updated at this new time, *t*, using the expressions:

$\xi_{i}(t)=p_{\xi_{i}}(t)+\Delta\xi_{i}$ *i* ⊂ *v_p_* and *ξ* = DRAQ5 and GFP [S3.2]

where $\Delta\xi_{i}$is the same as that defined in equation S3.1. To illustrate the evolution process, the solid black lines in Figure S5 indicate the 2D intensity trajectories of three randomly chosen *v*cells, from the virtual populous. These curves represent how these *v*cells (labelled by the yellow markers) and their subsequent off-spring, evolve through their intermitotic cell cycle unless perturbed by pharmacodynamic agents.

**S4 Cell population model – stochastic mitotic event**

This section describes the stochastic process by which the CPM determines if a *v*cell present in the 4N DNA index (G2/M) undergoes mitosis or evolves further in this phase. The CPM employs the stochastic decision similar to that detailed in [4] to determine whether or not a mitotic event as occurred. Each *v*cell has an associated with it a cumulative frequency distribution, CDF, the mean (and associated standard deviation) of which, corresponds to the mean intermitotic time (and its error) of the virtual population. These two parameters are to be optimized via the evolutionary algorithm (detailed in section S5) to best fit a second set of flow data obtained at a later time, *t*_1_. Once a *v*cell is deemed to have entered the 4N DNA phase (i.e. DRAQ(*t*) > DRAQ5_DNA2_) the associated CDF of that *v*cell at the time *t* is deduced producing a real number, R_CDF_, within the interval [0 1]. This CDF is then stochastically sampled by generating a random number, R_RND_, in the unit interval, if R_RND_ < R_CDF_ then the *v*cell undergoes a mitotic event, on the other hand if R_RND_ < R_CDF_ then the *v*cell remains in the 4N DNA phase and its intensity coordinates are updated accordingly. If the former is true then mitosis is deemed to occur and the CPM generates two daughter cells at the intensity coordinates DRAQ5(*t*_t=0_) and GFP(*t*_t=0_) deduced previously for the parent (see sub-section S3) and are left to evolve in time with the inherited properties: ΔDRAQ and ΔGFP (detailed in equation S3.1). Thus if the later is true at the subsequent time increment, this individual *v*cell will have an increased probability of undergoing mitosis due to the fact that its CDF is monotonically increasing to unity as a function of time.

**S5 Differential Evolution**

The second component of the simulation procedure is an evolutionary computing technique, named differential evolution (DE) [4]. Like most evolutionary algorithms, DE follows the traditional *modus operandi*: initialisation, mutation, selection and recombination. However, compared to other evolutionary strategies DE has many additional attractive characteristics: it employs a differential operator to create new candidate solutions, uses a one-to-one competition scheme to select new population members and naturally employs real numbers [5].

The cell population model is defined by a set of parameters specific to the flow cytometry experiment conducted. Optimization of the fit between simulation and experiment is dependent upon selection and minimization of the population variables, in our case: the mean inter-mitotic time, its standard deviation and a parameter detailing the presence of a drug in the *v*population. The upper and lower bounds for these optimisation parameters were set at 10-50 hours, 0-25 hours and 0-1 respectively.

The DE algorithm is initialised by randomly sampling from these bounds developing a population and assigning these three values as vector components in sample space. This process is repeated for the initial vector population of *N_p_* members. These vectors are then used to optimise the CPM optimisation parameters by searching the parameter space for a global minima related to the correspondence of the *v*population distribution in intensity space at a time t_1_, to that of a second flow Cytometric data set acquired also at t_1_. The correspondence or fitness the DE algorithm seeks, between the real and virtual data sets, is the maximisation of the ratio of *v*cells to that of the experimentally measured within a numerically defined gated area (see figures 5(a-c) of the manuscript for examples). Termination of the DE algorithm is determined when fitness magnitude varies by less than 1% over five subsequent DE generations.

**S6 Video - *v*population simulation**

(See movie attachment)

**S7 MATLAB code**

Below are examples of the functions used for the gating procedure detailed in S1 and the assignment of a temporal location through minimization detailed in S2-3. A diagrammatic illustration of the full numerical procedure is displayed in figure 2 of the manuscript.

***S.7.1 Gating***

function [x y xi yi] = clean_data(x,y,N,NpA)

% The function gating finds the experimental cells within a calculated

% contour or gating region, which is itself determined from the density of

% cells per unit elemental area.

% Inputs:

% x - DRAQ5 coordinates of vcells

% y - GFP coordinates of vcells

% NpA - cut-off parameter to inform elemental region is

% active or not

% Outputs:

% x - DRAQ5 coordinates of vcells within contour

% y - GFP coordinates of vcells within contour

% xi - DRAQ5 coordinates of contour

% yi - GFP coordinates of contour

%

% This function calls the following subfunctions:

% contourc - intrinsic MATLAB function

% inpolygon_mrb - modified intrinsic MATLAB function

maxv = max(max([x y],[],1));

minv = min(min([x y],[],1));

dI = (0:15:maxv)’;

N = numel(dI)

z = zeros(N,N);

% built 2D density map

for iy = 1:N-1

for ix = 1:N-1

xu = dI (ix+1); xl = dI (ix);

yu = dI (iy+1); yl = dI (iy);

counter = sum((x > xl & x <= xu) & (y > yl & y <= yu)) ;

if counter > NpA

z(ix,iy) = 1;

end

end

end

% Get 2 colour contours

N_c = 2;

C = contourc(dI, dI,z', NpA)';

isolines_xy = cell(N_c,1);

isolines_v = zeros(N_c,2);

i = 1;j = 1;

NelC = numel(C(:,1));

while j < NelC

isolines_v(i,:) = C(j,:);

idx = j+1:j+C(j,2);

isolines_xy{i,1} = C(idx,:);

j = idx(end) + 1;

i = i + 1;

end

isolines_v = isolines_v(1:i-1,:);

isolines_xy = isolines_xy(1:i-1,:);

% delete repeated contours

id = isolines_v(:,1) == NpA;

isolines_v = isolines_v(id,:);

isolines_xy = isolines_xy(id,:);

% locate longest contour

[isolines_v iu_isolines] = max(isolines_v(:,2));

isolines_xy = isolines_xy(iu_isolines,:);

% Get contour

xy = isolines_xy{1};

xi = xy(:,1); yi = xy(:,2);

% Get points on and within contour

in = inpolygon_mrb(x,y,xi,yi);

x = x(in); y = y(in);

***S.7.2 Temporal position in cell cycle***

function [t S] = temporal_position(S,x,y)

% The function temporal_position locates a relative temporal position in

% the cell% cycle for each *v*cell.

% Inputs:

% x - DRAQ5 coordinates of vcells

% y - GFP coordinates of vcells

% S – a structure whose entries contain *v*cell information see S2 and

% S3 for details

% Outputs:

% t – time in cell cycle

% S – a structure whose entries contain *v*cell information see S2 and

% S3 for details

%

% This function calls the following subfunctions:

% norm_renom_xy – (re)normalise intensity coordinates equation [S1.2.1]

% find_time – calculates equation [S2.3.1]

% xu_lmu_eval - calculates equation [S3.1-2]

% normalise data

[xn yn] = norm_renorm_xy(S,x,y,0);

t = rand(S.N,1,);

tol = 1e-8;

err = S.N;

it = 0;

% newton iteration

while err > tol && it < 1000

[f df] = find_time(S,xn,yn,t);

J = - f./(df);

t0 = t + J;

dt = (t0-t);

err = sum(dt.*dt)/S.N;

t = t0;

it = it + 1;

end

% find minimum of DRAQ and GFP signal to allow determination of G1S-G2M phase

S.dx = xu_lmu_eval(S,t,0,0);

S.dy = xu_lmu_eval(S,t,1,0);

% find minimum of DRAQ and GFP signal @ t = 0

t0 = 0;

S.x_t0 = xu_lmu_eval(S,t0,0,0);

S.y_t0 = xu_lmu_eval(S,t0,1,0);

% re-normalise data

[S.dxn S.dyn] = norm_renorm_xy(S,S.dx,S.dy,1);

S.t = t;

%%%%%%%%

**S8 References**

[1] Kaplan, E.L. & Meier, P. (1958). "Nonparametric estimation from incomplete observations". *Journal of the American Statistical Association* **53**: signal 457–481.

[2] <http://mathworld.wolfram.com/L2-Norm.html>.

[3] Kelley CT (2003) Solving Nonlinear Equations with Newton's Method. Society for Industrial Mathematics.

[4] Brown MR, Rees P, Wilks S, Summers HD, Errington RJ et al. (2007) Computational simulation of optical tracking of cell populations using quantum dot fluorophores. Lect. Notes Comput. SC, 4695:96-105.

[5] Price K and Storn R, Differential evolution homepage: [www.ICSI.Berkeley.edu/~storn/code.html.](../www.ICSI.Berkeley.edu/~storn/code.html.)

**Figure S1**: Flow cytometry data set indicating the DRAQ5 and GFP-cyclin B1 fluorescence intensities of a measured cell population. This plot highlights the raw data (grey) and the subsequent gated fraction (red); also, the three principle sub-fractions present within the raw data are numerically labelled.

**Figure S2**: Fluorescence intensity plot of the normalised *v*population; vcells highlighted in red refer to those with the lowest normalised DRAQ5 values.

**Figure S3**: Plot indicating the curve $p_{DRAQ}(t)$ v’s$p_{GFP}(t)$ over the cell cycle interval [0 IMT], and the *v*population, 3 members of which have been highlighted (yellow markers) with DRAQ5 and GFP intensity differences (red arrows) corresponding to equations [S2.3.2].

**Figure S4**: Figure indicating location of the roots (yellow markers) between the median intensity line and linear functions (that enclosed by red ellipses) describing evolution of DRAQ5 and GFP intensity coordinates as a function of time.

**Figure S5**: Intensity plot highlighting the properties of three randomly chosen vcells. These properties include: their vcells intensity coordinates at both t_E=0_ and t_t=0_ marked in yellow and red respectively and their corresponding intensity trajectory through their cell-cycle (solid black lines).
